# Insightful understanding of the correlations of the microstructure and catalytic performances of Pd@chitosan membrane catalysts studied by positron annihilation spectroscopy

**DOI:** 10.1039/c7ra12407d

**Published:** 2018-01-17

**Authors:** Qi Liu, Mengdie Xu, Jing Zhao, Yudong Wang, Chenze Qi, Minfeng Zeng, Rui Xia, Xingzhong Cao, Baoyi Wang

**Affiliations:** Zhejiang Key Laboratory of Alternative Technologies for Fine Chemicals Process, Shaoxing University Shaoxing 312000 China zengmf@usx.edu.cn; Institute of High Energy Physics, The Chinese Academy of Science Beijing 100049 China caoxzh@ihep.ac.cn

## Abstract

In this study, the catalytic performances of palladium supported on chitosan (Pd@CS) membrane heterogeneous catalysts have been studied from the aspects of free volume by positron annihilation lifetime spectroscopy (PALS). The results showed that the variation in free volume hole size of the Pd@CS membrane catalyst was closely associated with microstructure evolutions, such as increase of Pd content, valence transition of Pd by reduction treatment, solvent swelling, physical aging during catalyst recycling, and so on. The PALS results showed that both the mean free volume hole size of the Pd^0^@CS membrane in the dry or swollen state (analyzed by the LT program) and its distribution (analyzed by the MELT program) are smaller than the molecule size of the reactants and products in the catalysis reaction. However, the results showed that the Pd^0^@CS membrane catalyst has excellent catalytic activity for the Heck coupling reaction of all the reactants with different molecule size. It was revealed that the molecule transport channels of the Pd^0^@CS membrane catalyst in the reaction at high temperature was through a number of instantaneously connected free volume holes rather than a single free volume hole. This hypothesis was powerfully supported by the catalytic activity assessment results of the CS layer sealed Pd^0^@CS membrane catalyst. Meanwhile, it was confirmed that the leaching of Pd^0^ nanoparticles of the reused Pd^0^@CS membrane catalyst during the recycling process was also through such instantaneously connected free volume holes.

## Abstract 2

A number of instantaneously connected free volume holes act as mass transport channels of the Pd^0^@CS membrane catalyst in reactions.

## Graphical abstract

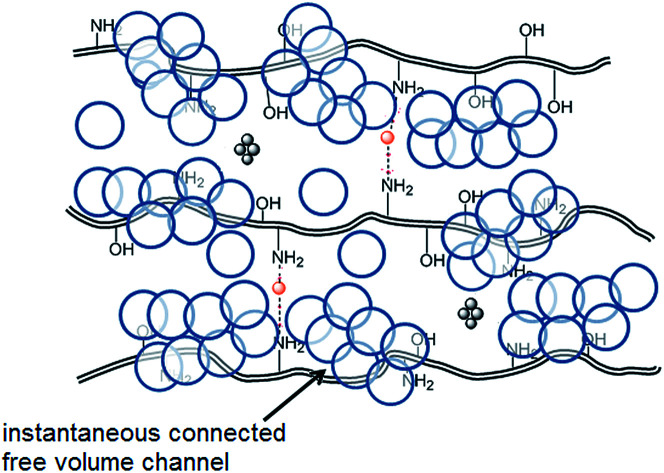

## 1. Introduction

Transition metals, such as palladium, rhodium, ruthenium, and other precious metals, are frequently used as efficient catalysts in organic synthesis reactions. Conventionally, the transition metal catalysis process is often employed in homogeneous systems for its excellent activity and selectivity.^1^ However, in homogeneous catalysis, it is not easy to separate, recover, and reuse the high cost transition metals.^2^ From the point of view of environmental protection and economics, the applications of homogeneous catalysis in industry are limited. In order to reduce the drawbacks of homogeneous catalysis, the development of heterogeneous catalysis by immobilization of transition metal catalysts on a solid matrix have attracted more and more attention in recent years.^3–5^ Immobilization of transition metal catalysts on a solid matrix can effectively reduce the drawbacks of homogeneous catalysis. Also, it can offer a number of advantages, such as ease of handling, product isolation, recovery/recycling of transition metal catalysts, obtaining high purity of products without contamination of transition metals, and so on. Therefore, the preparation of transition metal heterogeneous catalysts by immobilization of transition metal on inert materials or functional solid materials with high activity, good selectivity, and excellent stability/recyclability, has been one of the hotspots in green and sustainable chemistry.^6^ Inorganic materials, like zeolites,^7–11^ alumina,^12–15^ silica,^16–18^ and clay,^19–21^ are commonly used supports for the preparation of supported transition metal catalysts. However, the inorganic carries often need modification to further improve their chelating ability with transition metal catalysts. Recently, more and more attentions has been paid to bio-polymeric supports, which have strong chelating ability with transition metals for the containing of numerous polar groups on the macromolecule backbone, especially carbohydrate polymers, such as starch,^22,23^ cellulose,^24–26^ alginate,^27,28^ and chitosan,^29–33^*etc.* Among them, chitosan (CS), deacetylated products of chitin, is considered to be one of the most promising transition metal catalysts supports for its high chelating ability with the transition metals (containing plenty of amino-groups and hydroxyl groups on the macromolecule backbone) and easy-tailoring of processing into different forms including membranes, microspheres, and nanofibers, *etc.* Hence, numerous studies have highlighted the application of transition metal supported on CS-based materials in catalysis.^34,35^

It is well known that, well-defined porous structure with high specific surface areas is generally required for an efficient heterogeneous catalyst. Porous structure with high specific area is beneficial to a heterogeneous catalyst exhibiting good mass transport performance for organic molecules. Many porous forming techniques, including porogen leaching,^36,37^ supercritical CO_2_ technique,^38^ freeze drying,^39^*etc.*, have been used in preparation of porous CS-based materials. However, the introduction of open porous structure is often disadvantageous to the stability of the polymer supports under harsh reaction conditions (such as high reaction temperature, continuous processing, and swelling of the solvents, *etc.*), resulting in limited recycling performance of the heterogeneous catalyst. Recently, many works^40–43^ have been reported on excellent palladium heterogeneous catalysts by directly confining Pd nanoparticles in polymer matrix. The prepared palladium heterogeneous catalysts have neither open porous structure nor high specific areas, but they have similar activity and higher stability as compared with general prepared palladium supported on porous polymer supports heterogeneous catalysts. As we know, molecules permeation through polymer membranes (with no visible pores) has been traditionally described by the solution-diffusion model. In solution-diffusion model, enough spaces between the polymer chains are needed for the molecules to permeate into and diffuse through the polymer membrane. The good performances should be mainly attributed to the sub-nano scale open spaces (*i.e.* free volume holes) within swollen polymer matrix, which might allow the reactants and products molecules to get access to the well-entrapped Pd nanoparticles during reactions. As reported by Dong *et al.*,^44^ the mesh size of the crosslinked polyvinyl alcohol (PVA) and polyacrylamide (PAM) estimated by swelling method was 4.0 nm and 2.6 nm, respectively, which was large enough for the diffusion of the reactants and products molecules as applied in Heck coupling reactions. As determined by positron annihilation spectroscopy, our initial study^41^ showed that the mean diameter of the free volume holes of swollen Pd@CS membrane catalyst ranged from 0.5 to 1.2 nm, which is close to the size of the reactants as applied in Heck reaction. Moreover, previous studies^45–50^ have demonstrated the free volume properties of the polymer membrane was the governing factor controlling the transport performances of gas (CO_2_), water, solutes, and so on. Therefore, it is believed that the free volume holes of the polymer membrane could be the diffusion channels for the reactants. And it is of interest and importance to study the correlations of the microstructure and catalytic performances of Pd@CS membranes. This prompted an additional and thorough research into the governing mechanisms at molecular level behind the Pd@CS membrane catalyst, which is reported in this study.

However, there are few techniques capable of characterization of the microstructure of polymer membranes in sub-nanometer level. Surface characterization techniques, such as transmission electron microscopy (TEM) and scanning electron microscopy (SEM), are unable to determine the free volume holes of polymer materials. Other methods, such as N_2_ adsorption–desorption methods and differential scanning calorimeter (DSC) are not suitable and/or do not have adequate sensitivity to quantify the hole size in sub-nanometer scale. Though the swelling method is convenient, it is not a direct method and does not have enough accuracy in the results. Never mind, positron annihilation lifetime spectroscopy method has allowed sensitive characterizations of the internal microstructure of polymers in sub-nanometer level. An increasing number of PALS studies of polymer membrane characterization have been conducted over the last few years.^51–54^ It can directly provide information about the free volume properties of polymers, such as free volume hole size, distribution, and free volume fraction. In polymers, a part of the implanted positrons (e^+^) will combine the electrons (e^−^) from surrounding molecules to form positronium (Ps) atoms. Ps has two spin states: *para*-positronium (*p*-Ps, the spins are antiparallel) and *ortho*-positronium (*o*-Ps, the spins are parallel). Under vacuum conditions, the intrinsic of *o*-Ps lifetime is 142 ns *via* 3γ annihilation. In polymers, *o*-Ps is localized in free volume holes, and its lifetime is shortened down to a few ns *via* 2γ pickup annihilation. In general, three annihilation processes are possible in polymers: *p*-Ps annihilation (lifetime *τ*_1_, about 0.125 ns), free positron annihilation (lifetime *τ*_2_, about 0.4 ns), *o*-Ps annihilation (lifetime *τ*_3_, 1–10 ns). The mean free-volume hole radius (*R*) of polymers is determined by its relationship with the longest *o*-Ps lifetime (*τ*_3_) given by Tao–Eldrup model^55,56^ as shown in eqn (1),1
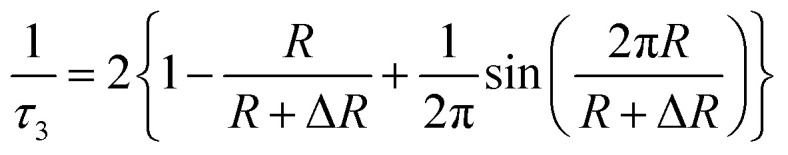
where Δ*R* is the fitted empirical electron layer thickness, Δ*R* = 0.1656 nm. And the mean free volume hole diameter, *D*, can be calculated according to the eqn (2).

2 *D* = 2*R*

It is noteworthy that the catalytic mechanism of transition metals supported on polymer membranes has rarely studied from the aspects of free volume. In this study, the main purpose was to provide a comprehensive and insightful understanding on the catalytic mechanism of Pd@CS membrane heterogeneous catalysts, particular focusing on free-volume properties correlations with the molecules transport behavior and physical aging behavior during the catalyst recycling. Other characterization methods, such as high resolution transmission electron microscopy (HR-TEM), X-ray photoelectron spectroscopy (XPS), thermal gravity analysis (TGA), inductively coupled plasma-atomic emission spectroscopy (ICP), *etc.*, have been used to characterize the microstructure of the prepared Pd@CS membrane heterogeneous catalysts.

## 2. Experimental

### 2.1 Materials

Chitosan, 95% deacetylated, with a viscosity average molecular weight of 1.2 × 10^5^, was purchased from Zhejiang Aoxing Biotechnology Co., Ltd., China. PdCl_2_ raw materials were purchased from Zhejiang Metallurgical Research Institute Co., Ltd., China. The aromatic halides reagents were purchased from Energy Chemical, Sun Chemical Technology (Shanghai) Co., Ltd. The other chemical reagents and solvents were purchased from by Sinopharm Chemical Reagent Co., Ltd.

### 2.2 Preparation of Pd@CS membrane catalyst

The preparation process of the common Pd@CS membrane catalyst was as follows. Under magnetic stirring, 1 g of CS was dissolved in 100 ml of 2 wt% acetic acid solution. Na_2_PdCl_4_ solution was prepared by dissolving of 0.15 g of PdCl_2_ in 50 ml of 2 wt% NaCl solution. 0, 2.5, 5, and 7.5 ml of Na_2_PdCl_4_ solution were dropped into the CS solution and magnetically stirred for another 0.25 h. The resultant gel solution was then casted on PTFE dishes, and was dried at 60 °C for about 4 h to form membranes. The membranes were immersed in 5 wt% NaOH bath for 0.5 h to remove the remaining acid. Then, the membranes were washed with deionized water to pH = 7. After drying at 60 °C, the Pd^2+^@CS membranes were prepared. The resultant Pd^2+^@CS membranes were further reduced to Pd^0^@CS membranes by immersing into 80 °C ethylene glycol bath for about 0.5 h. The color of the Pd^0^@CS membranes is much ducker than Pd^2+^@CS membranes, confirming the formation of Pd^0^ nanoparticles. The resulting Pd@CS membranes with different Pd addition amounts (2.5, 5, and 7.5 ml of Na_2_PdCl_4_ solution) were labeled as Pd@CS-1, Pd@CS-2, and Pd@CS-3, respectively.

**Scheme 1:**
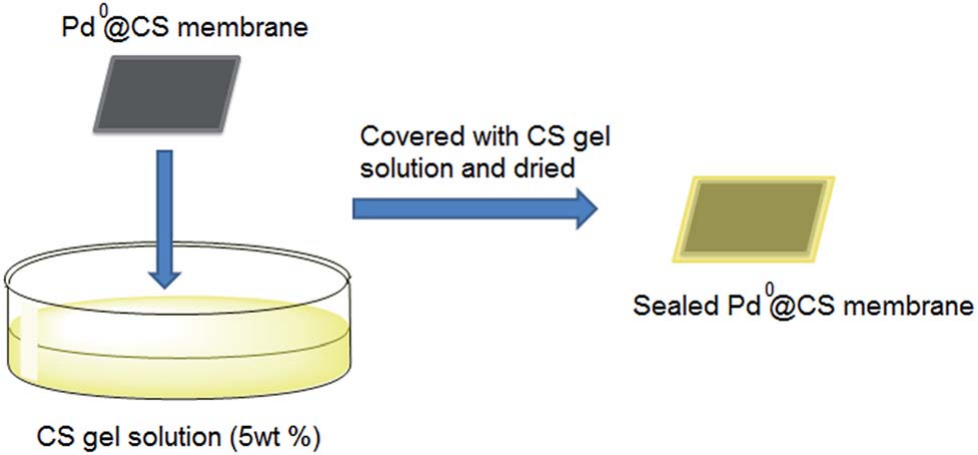
The preparation of CS layer sealed Pd^0^@CS membrane catalyst.

To deep assess the molecule transport behavior of the Pd^0^@CS membrane catalysts, we also prepared a number of CS layer sealed Pd^0^@CS membrane samples. The preparation process of the CS layer sealed Pd^0^@CS membrane samples were illustrated as Scheme 1. The resultant Pd^0^@CS membrane catalysts were coated completely with CS gel solution with high concentration (5 wt%). After drying at 60 °C, the common Pd^0^@CS membrane catalysts were then sealed completely with CS layers.

### 2.3 Catalytic application of Pd@CS membrane catalyst

In a 50 ml round bottom flask, a mixture of aryl iodide (1 mmol), acrylate (2 mmol), Pd@CS-3 membrane catalyst (0.005 mmol), CH_3_COOK (3 mmol), solvents (10 ml of dimethyl sulfoxide (DMSO) + 0.2 ml ethylene glycol) was stirred at 110 °C (heated in oil bath) for 5 h. The progress of the reactions was monitored by thin layer chromatography (TLC) and/or gas chromatography-mass spectrometry (GC/MS) analysis. The chemical structure and yield of the coupling reaction products were confirmed using ^1^H NMR and GC/MS analysis. The structure characterization results of the coupling products were consistent with our recent work.^57^ In recycling experiments, the filtered Pd^0^@CS membrane catalysts were rinsed with ethanol firstly, and then they were dried and reused in a second reaction run.

### 2.4 Characterization of Pd@CS membrane catalyst

The mechanical properties of Pd@CS membrane catalysts were measured with a SANS universal materials testing instrument (Shenzhen SANS testing machine Co. Ltd., China) according to GB/T 1040.3-2006 (standard test method for tensile properties of plastic film of China), and the testing condition was, at 25 °C, with a crosshead speed of 50 mm min^−1^.

The swelling ratio of the Pd@CS membrane catalysts in ethylene glycol and DMSO was determined as follows. The pre-weighed Pd@CS membrane samples were immersed in ethylene glycol or DMSO for 1 h at 110 °C with magnetic stirring. Then, the membrane samples were taken out of the solvents. The samples were weighed again after removing the excess solvents on the surface of the samples by tissue. The swelling ratio (*S*) of the membrane samples was calculated with eqn (3)

3 *S* = (*m*_t_ − *m*_0_)/*m*_0_ × 100%

where *m*_t_ and *m*_0_ are the weights of the membrane samples after and before immersion in the solvents.

The thermal stability of the Pd@CS membrane catalysts was measured with a Mettler Toledo TGA/DSC 2 STAR^e^ system, and the testing condition was: air atmosphere, from 30 to 800 °C, at a scanning rate of 20 °C min^−1^.

The crystalline structure of Pd@CS membrane catalysts was measured with an Empyrean X-ray diffraction system and the testing condition was: diffraction angle 2*θ* from 3° to 70°, at a scanning rate of 2° min^−1^.

The binding energy of Pd was measured by a Thermo Scientific ESCALAB 250Xi X-ray photoelectron spectrometer. The morphology observation was performed with JEM-2100F high resolution transmission electron microscope (HR-TEM).

Pd contents within Pd@CS membrane catalyst were determined with a Leemann ICP-AES Prodigy XP inductively coupled plasma atomic emission spectrometer.

Positron annihilation lifetime measurements of the Pd@CS membrane samples were performed using an EG&G ORTEC fast-slow system, with a time resolution of about 210 ps (full width at half-maximum, FWHM). The positron source (^22^Na, 16 μCi) was NaCl (carrier free) which was deposited between two Kapton foils (7 μm in thickness). And then it was sandwiched in two stacks of identical pieces of Pd@CS membrane samples (1 cm in width, 1.5 mm in thickness). The total counts of the positron lifetime spectra of each sample were at least 3.5 × 10^6^ to achieve good statistics. Both LT-9 (Lifetime-9) program and MELT-4 (Maximum Entropy for Lifetime Analysis-4) programs were used to analyze the positron annihilation lifetime spectra with three components fitting.

## 3. Results and discussion

**Fig. 1:**
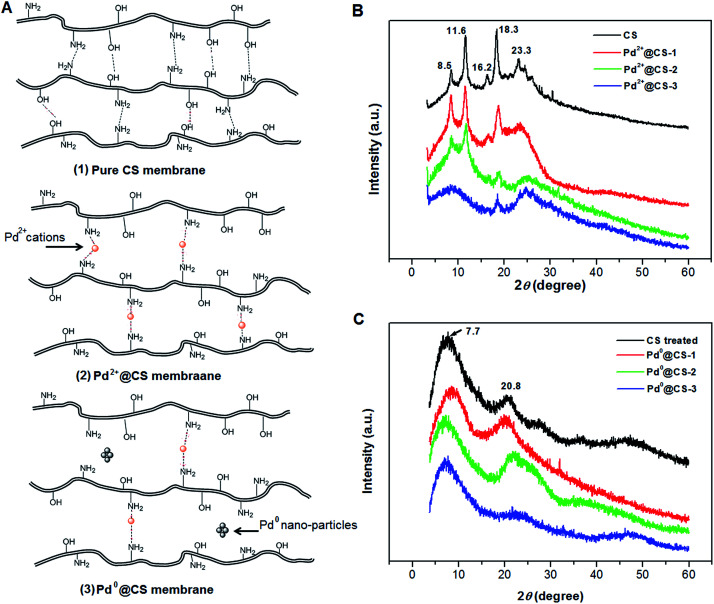
The molecule structure and XRD patterns of pure CS and prepared Pd@CS membranes: (A) molecule structure of the Pd@CS membranes; (B) Pd^2+^@CS membranes (before reduction), (C) Pd^0^@CS membranes (after reduction).

The molecule structure and crystalline structure of pure CS and prepared Pd@CS membranes are described in Fig. 1. For pure CS membrane, during the membrane preparation, strong inter-macromolecular hydrogen bonds have formed between the polar groups (–NH_2_ and/or –OH groups) of the macromolecules (Fig. 1A(1)). Pure CS membrane shows five diffraction peaks at 2*θ* of 8.5°, 11.6°, 16.2°, 18.3°, and 23.3°, respectively, indicating tight and regular packing of CS macromolecules. In the case of Pd^2+^@CS membranes, the added Pd^2+^ cations act as crosslinking points for CS macromolecules by their complex reactions mainly with the –NH_2_ groups (Fig. 1A(2)). As shown in Fig. 1B, the corresponding diffraction peaks of CS become much weaker and broader as the Pd^2+^ content increases, indicating the decrease in regularity packing of CS macromolecules. The reason should be due to the replacements of the hydrogen bonding by the Pd^2+^ induced crosslinking of the CS macromolecules. It is well known that Pd^2+^ cations are easily reduced by reductively solvents, like alcohols, DMSO, *etc.*^32,33^ The Pd^2+^@CS membranes were then reduced by ethylene glycol to prepare Pd^0^@CS membrane catalysts (Fig. 1A(3)). Same treatment was done to the pure CS membrane for comparing. As shown in Fig. 1C, the treated CS membrane shows only two broad diffraction peaks at 7.7° and 20.8°. It means that the tightness and regularity of CS macromolecules packing decreases obviously after the solvent permeation into the space of the CS macromolecules. Similar phenomenon is found for the Pd^0^@CS membrane catalyst prepared by the reduction of Pd^2+^@CS membrane with ethylene glycol. However, no obvious characteristic diffraction peaks for Pd^0^ nano-particles were detected, suggesting that both the content and size of the Pd^0^ nano-particles were below the detection limit of XRD.

**Fig. 2:**
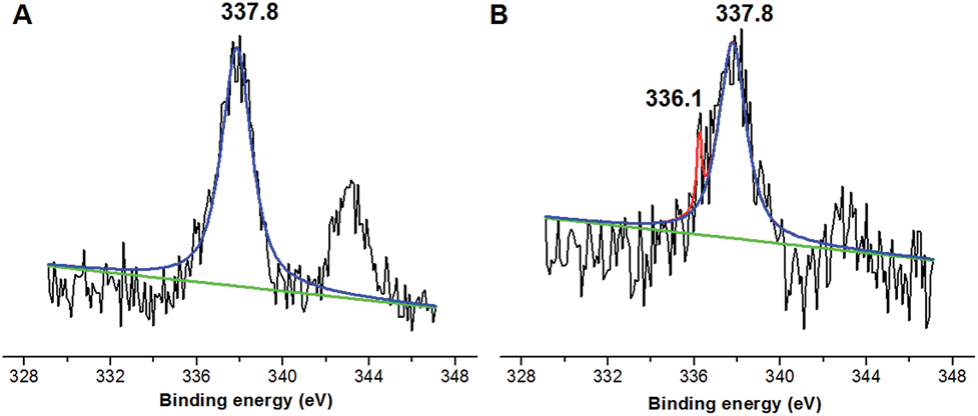
X-ray photoelectron spectra of (A) Pd^2+^@CS-3 and (B) Pd^0^@CS-3 membrane catalyst.

**Fig. 3:**
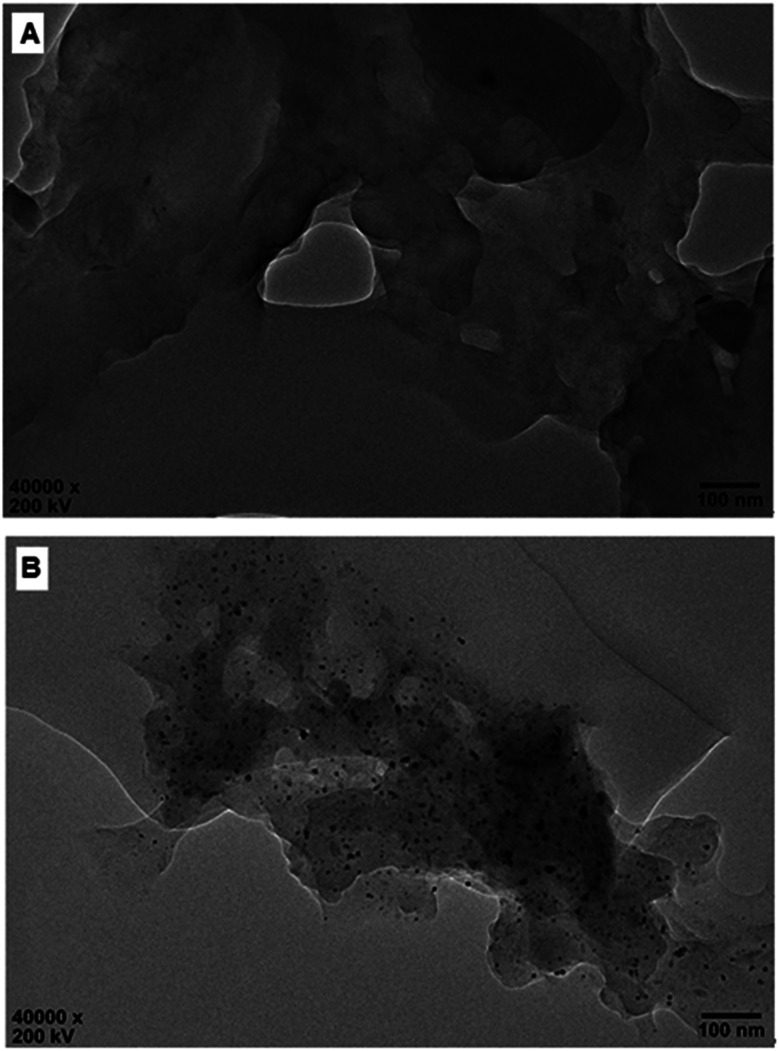
HR-TEM observation of (A) Pd^2+^@CS-3 and (B) Pd^0^@CS-3 membrane catalyst.

Nevertheless, the formation of Pd^0^ nano-particles can be well demonstrated by XPS characterization and HR-TEM observation. Fig. 2A shows the fresh Pd^2+^@CS-3 membrane has the characteristic Pd 3d_5/2_ electron binding energy peak at 337.8 eV, which is identical to that of the divalent palladium,^58^ confirming the Pd species in the fresh Pd^2+^@CS-3 membrane is in divalent state. In the case of Pd^0^@CS-3 membrane, as shown in Fig. 2B, besides the characteristic Pd 3d_5/2_ electron binding energy peak at 337.8 eV, a new peak at lower electron binding energy at 336.1 eV is detected. Although the peak at 336.1 is a bit weak in intensity, the appearance of this peak indicates that some of the Pd^2+^ species is reduced to Pd^0^ species.^58^ The Pd@CS-3 samples were embedded in epoxy resin, then microtomed, and finally imaged by HR-TEM. As shown in Fig. 3A, no individual separated Pd species are observed for the fresh Pd^2+^@CS membrane, indicating the added Pd^2+^ cations dispersed in molecular level in CS matrix. After reduction of Pd^2+^ to Pd^0^ by ethylene glycol, plenty of Pd^0^ nano-particles sized in about 5 nm are visualized in the CS matrix (Fig. 3B). It reveals that Pd^0^ species has less miscibility with CS matrix and form nano-sized aggregates dispersed in CS matrix.

The mechanical properties and swelling behavior of the Pd^0^@CS membrane catalyst are summarized in Table 1. Clearly, the Pd^2+^-induced crosslinking is advantageous for the improvement of the mechanical properties. For example, the tensile strength and modulus of the Pd^0^@CS-3 membrane catalyst are 24.9 MPa, and 619 MPa respectively, which is much higher than pure CS membrane. The equilibrium swelling ratio of the Pd^0^@CS membrane in solvents were determined as 56–62% in DMSO, and 89–91% in ethylene glycol. It is reasonable that high Pd contents and mechanical properties are in favor of high activity and stability in catalysis reactions. Therefore, in this study, we mainly focus on the Pd^0^@CS-3 membrane catalyst for its highest Pd content and mechanical properties.

**Table 1:** Mechanical properties and swelling ratio of the Pd^0^@CS membranes with different Pd contents

Samples	Tensile modulus (MPa)	Tensile strength (MPa)	Swelling ratio (%)	
			DMSO	Ethylene glycol
CS	237	11.8	57	86
Pd^0^@CS-1	311	17.6	58	90
Pd^0^@CS-2	472	22.3	56	89
Pd^0^@CS-3	619	24.9	62	91

**Fig. 4:**
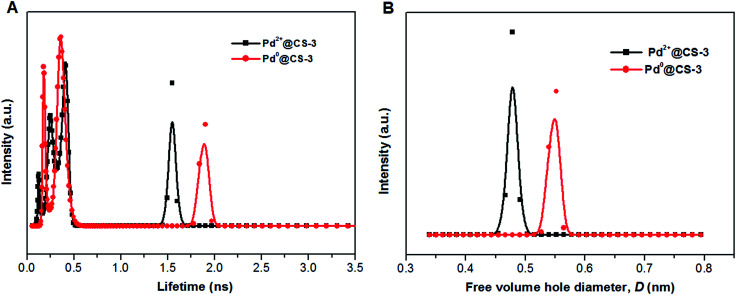
The distribution of positron annihilation lifetime (A) and free volume hole size of the Pd@CS-3 membrane (B) analyzed by MELT program.

To obtain an in-depth understanding of the local environment of Pd species in CS matrix and the mechanism of the heterogeneous catalysis, PALS was applied as a powerful method to directly provide information on the micro-voids of the Pd@CS membrane with the resolution at molecular level. Firstly, the LT-program was used to fit the PALS spectra to evaluate the mean value of lifetimes and intensities. As shown in Table 2, the PALS spectra was fitted well in three-component lifetimes, *p*-Ps annihilation and free positron annihilation (shortest lifetime component, *τ*_1_, and its intensity, *I*_1_), free positron annihilation and positron trapping in the interfaces of CS and Pd species (intermediate lifetime component, *τ*_2_, and its intensity, *I*_2_), and *o*-Ps annihilation (longest lifetime component, *τ*_3_, *I*_3_). The free volume hole sizes in CS and Pd@CS membrane were then calculated by eqn (1) and eqn (2) from *o*-Ps lifetime, *τ*_3_. For pure CS membrane, the free volume hole diameter, *D* value was calculated as 0.5194 nm. With addition of Pd^2+^, it is found that the *D* value increases with the increase of the Pd^2+^ content. The *D* value of Pd^2+^@CS-1, Pd^2+^@CS-2, and Pd^2+^@CS-3 are 0.5424, 0.5466, and 0.5558 nm, respectively. It indicates that the open space between the CS macromolecules is enlarged with addition of Pd^2+^. For pure CS membrane, the main inter-macromolecular force is inter-macromolecular hydrogen bonding, which is formed by the directly interactions between the polar groups of neighboring CS macromolecules. In the case of Pd^2+^@CS membrane, the neighboring CS macromolecules are connected together by crosslinking points of Pd^2+^ cations. Therefore, it is reasonable that the open space between CS macromolecules will be enlarged for the introduction of crosslinking points of Pd^2+^ into CS inter-macromolecules. The effects of the reduction treatment of the membrane with ethylene glycol on the *D* value were also evaluated. It is found that the *D* value of pure CS membrane increases from 0.5194 to 0.6098 nm after treatment with ethylene glycol, suggesting that the open space between CS macromolecules is enlarged effectively after the entrance of the polar solvent of ethylene glycol. More interactions are formed between CS macromolecules and polar solvent of ethylene glycol. Therefore, the compactness and regularity of CS molecular chain segments stacking decrease obviously. This result is consistent with the results of XRD. Similarly, the free volume size of Pd^0^@CS membrane is bigger than Pd^2+^@CS membrane for the reduction treatment with ethylene glycol. The *D* value of Pd^0^@CS-1, Pd^0^@CS-2, and Pd^0^@CS-3 are 0.6046, 0.5998, and 0.5808 nm, respectively. For the Pd^0^@CS membranes, the *D* value decreases with the increase of the Pd content. It should be related to the crosslinking degree of Pd^0^@CS membranes. Pd^0^@CS membrane with higher Pd content has higher resistance to the solvents for its higher crosslinking degree. The MELT-4 program was further used to fit the PALS spectra of Pd@CS-3 to evaluate the distribution of the lifetimes. As shown in Fig. 4, for Pd^2+^@CS-3, the longest lifetime component should be attributed to the *o*-Ps annihilation in the Pd^2+^-crosslinked CS matrix. The distribution range of *D* value of Pd^2+^@CS-3 was estimated to be 0.44–0.51 nm calculated by eqn (1) and (2) from the *o*-Ps lifetime. The increase of the free volume hole size after reduction treatment of Pd^2+^ to Pd^0^ was also sensitively detected by PALS fitted with MELT-4 program. For Pd^0^@CS-3, the distribution of *D* value was estimated to be 0.51–0.58 nm.

**Table 2:** Variation of positron annihilation lifetimes, intensities, and mean diameter of free volume holes of the Pd@CS membranes analyzed by LT-9 program

Samples	*τ*_1_ (ns)	*I*_1_ (%)	*τ*_2_ (ns)	*I*_2_ (%)	*τ*_3_ (ns)	*I*_3_ (%)	*D* (nm)
CS	0.1899 ± 0.0036	50.2 ± 1.0	0.4465 ± 0.0083	38.4 ± 1.0	1.733 ± 0.009	11.4 ± 0.3	0.5181 ± 0.0018
Pd^2+^@CS-1	0.1930 ± 0.0034	51.1 ± 1.1	0.4345 ± 0.0082	39.5 ± 1.1	1.855 ± 0.012	9.4 ± 0.2	0.5424 ± 0.0023
Pd^2+^@CS-2	0.1963 ± 0.0034	54.0 ± 1.1	0.4414 ± 0.0077	37.5 ± 1.1	1.877 ± 0.015	8.5 ± 0.2	0.5466 ± 0.0029
Pd^2+^@CS-3	0.1977 ± 0.0032	56.0 ± 1.1	0.4428 ± 0.0082	36.5 ± 1.1	1.925 ± 0.015	7.5 ± 0.2	0.5558 ± 0.0028
CS treated	0.1841 ± 0.0034	46.9 ± 1.1	0.4308 ± 0.0073	39.7 ± 1.0	2.228 ± 0.010	13.4 ± 0.3	0.6098 ± 0.0016
Pd^0^@CS-1	0.1782 ± 0.0036	43.9 ± 1.1	0.4019 ± 0.0063	44.5 ± 1.1	2.198 ± 0.012	11.6 ± 0.3	0.6046 ± 0.0021
Pd^0^@CS-2	0.1780 ± 0.0039	44.8 ± 1.1	0.4000 ± 0.0058	44.9 ± 1.1	2.170 ± 0.013	10.3 ± 0.2	0.5998 ± 0.0023
Pd^0^@CS-3	0.2007 ± 0.0037	54.9 ± 1.2	0.4042 ± 0.0078	38.5 ± 1.2	2.061 ± 0.013	6.6 ± 0.2	0.5808 ± 0.0023

Pd^0^-catalyzed Heck coupling reaction is one of the most powerful and versatile methods to construct new C–C bonds linked to unsaturated double bonds. In this study, Heck coupling reactions between aryl iodides and acrylates with different molecule size has been catalyzed by Pd^0^@CS-3 and CS layer sealed Pd^0^@CS-3. The main purpose is to assess the probabilities of the free volume holes as the molecules transport channels for the reactions. It should be noted here that, due to the swelling effects of solvent (DMSO swelling) and high temperature condition (110 °C), the open spaces between the CS macromolecules of the Pd^0^@CS-3 membrane catalyst in real reaction application should be enlarged much as compared to its dry state.^59–61^ Therefore, an approximate evaluation of the free volume hole diameter of the swollen Pd^0^@CS membrane in the reaction system was done firstly. Considering the swelling effect of solvent to the membrane catalyst, the free volume hole diameter of swollen Pd^0^@CS-3 membrane in the reaction system might be adjusted with the following eqn (4).^41^

4 *D*_s_ = *D* × (100% + swelling ratio)

where *D*_s_ is the mean free volume hole diameter of swollen membrane in the reaction system; *D* is the mean free volume hole diameter of dry membrane (as shown in Table 2); swelling ratio in DMSO is shown in Table 1. The *D*_s_ value of Pd^0^@CS-3 membrane was then estimated to be 0.9409 nm.

**Scheme 2:**
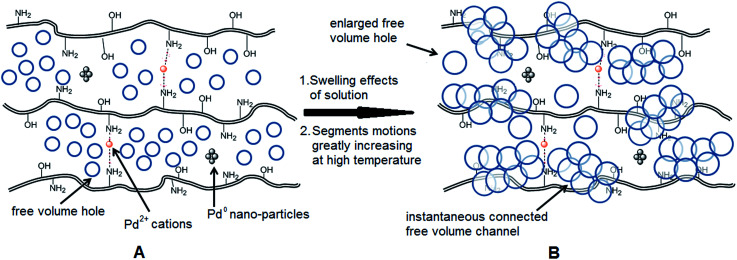
Diagrams of the formation of instantaneously connected free volume channels of Pd^0^@CS-3 membrane catalyst applied in Heck reactions: (A) fresh prepared Pd^0^@CS-3 membrane catalyst; (B) swollen Pd^0^@CS-3 membrane catalyst.

As we know, for Pd^0^@CS membrane catalyst, the Pd^0^ nanoparticles were well entrapped in the CS matrix. It is believed that the coupling reactions can occur only when the reactants molecules are able to diffuse into the Pd^0^@CS-3 membrane and get access to the nano-Pd^0^ catalyst. Therefore, the size of the open spaces of the membrane catalyst should be at least bigger than that of the involving molecules of the reactions. As shown in Table 3, for comparing, the mean *D*_s_ value of Pd^0^@CS-3 membrane (0.9409 nm) is bigger than the molecule size of the reactants of iodo benzene, iodine naphthalene, and *tert*-butyl acrylate; a bit smaller than that of substrates of iodine fluorine, and *n*-butyl acrylate; and much smaller than that of all the products molecules. It seems that the catalytic activity of the Pd^0^@CS-3 membrane catalyst should not be so good for the coupling reaction of these reactants. However, the reaction results show that the Pd^0^@CS-3 membrane catalyst has high catalytic activity for all the substrates with different molecule size. It revealed that many open spaces with big enough size should be induced within the CS matrix during the reaction to allow the reactants molecules and the products molecules diffusing freely in the membrane. To test this hypothesis, the Pd^0^@CS-3 membrane catalyst was further sealed with pure CS coating layer and applied in the same Heck coupling reactions. As shown in Table 3, although the coupling yields of all entries (especially in the entries of 2, 3, 5, 6 of big-sized reactants) are reasonably reduced obviously, the Heck coupling reactions of each entry can still be catalyzed by such CS layer sealed Pd^0^@CS-3 membrane catalyst, indicating that the involving molecules of reactions are able to diffuse through the open spaces of the CS layer. These results powerfully demonstrate that the open spaces of Pd^0^@CS-3 membrane catalyst should be enlarged enough for molecules transport during the reactions. The molecules transport channels of Pd^0^@CS membrane catalyst in the reaction should be through a number of instantaneously connected free volume holes rather than a single free volume hole. The catalysis mechanism of the Pd^0^@CS-3 membrane catalyst from the aspect of free volume could be illustrated in Scheme 2. On the one hand, when applied in Heck reactions, the swelling effects of the solvent to the Pd^0^@CS-3 membrane catalyst will lead to an increase of the open spaces between CS macromolecules. As a result, the free volume hole size of the swollen Pd^0^@CS-3 membrane is enlarged to some extent (as shown in Scheme 2B). On the other hand, the movement ability of CS segments of the Pd^0^@CS-3 membrane catalyst will be improved remarkably in the reaction system at high temperature (110 °C). Meanwhile, a number of neighboring free volume holes might undergo instantaneously connected during the segments motions to form much enlarged free volume hole channels (as shown in Scheme 2B), which provides enough space for substrates and the coupling products molecules diffusing freely in the membrane catalyst.

**Table 3:** Heck coupling reactions between aryl iodides and acrylates catalyzed with (A) Pd@CS-3 membrane and (B) CS layer sealed Pd@CS-3 membrane catalysts^a^

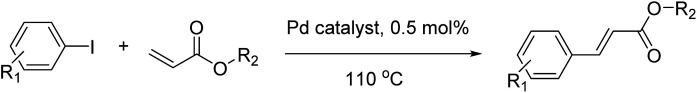					
Entry	Aryl iodide	Acrylates	Products	Yield^b^ (%)	
				A	B
1	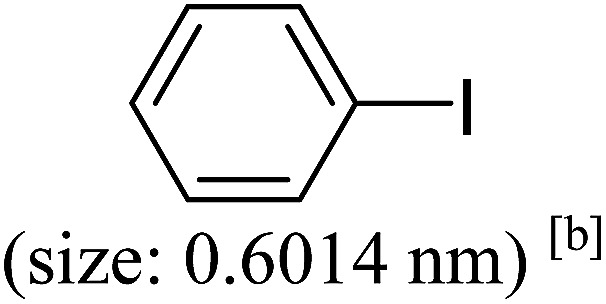	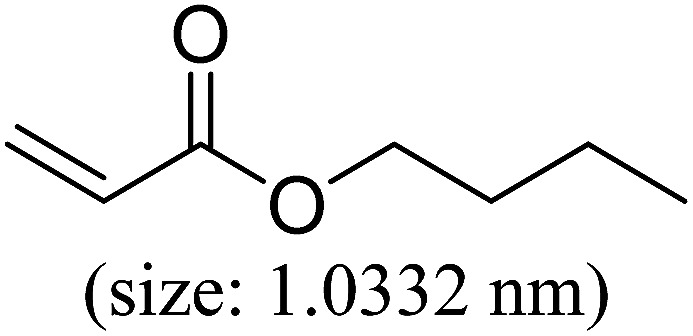	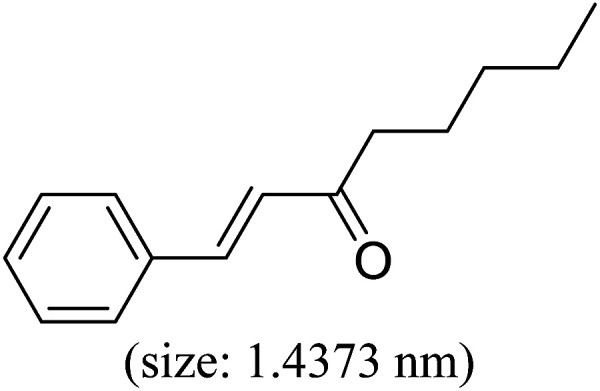	99	74
2	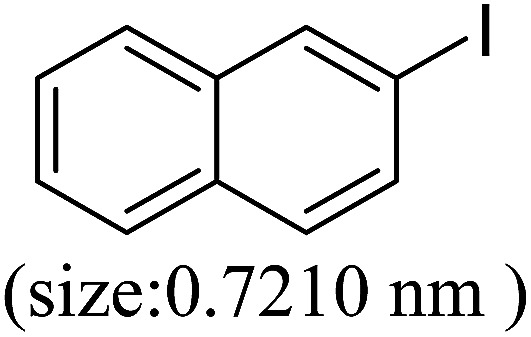	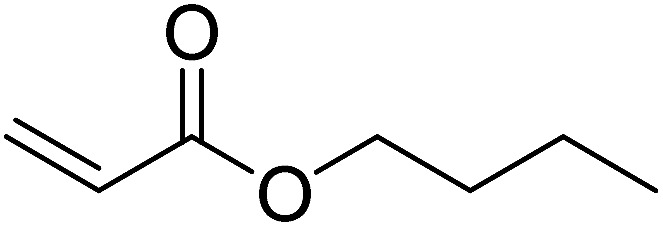	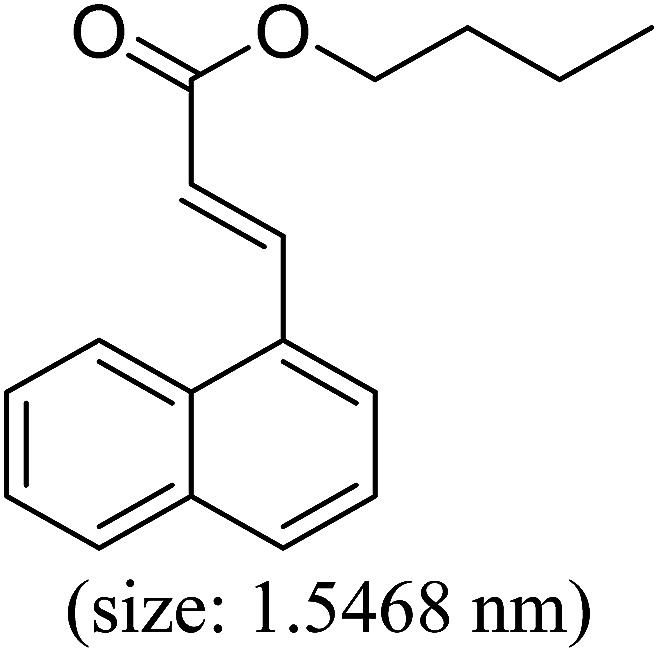	87	48
3	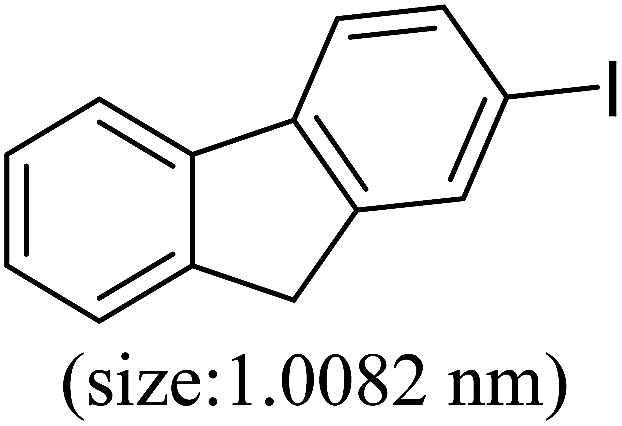	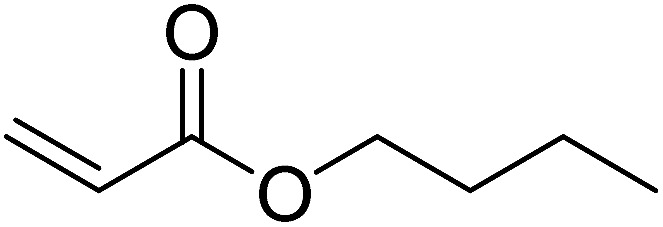	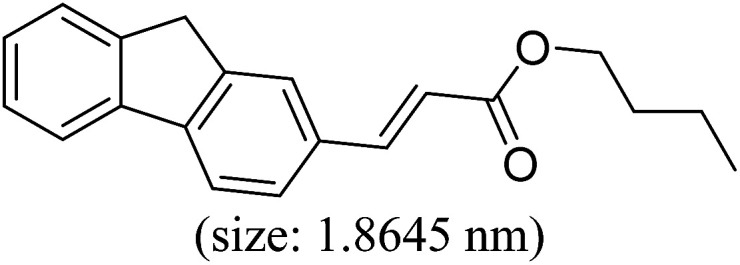	86	35
4	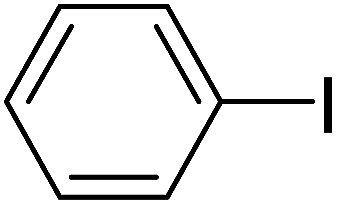	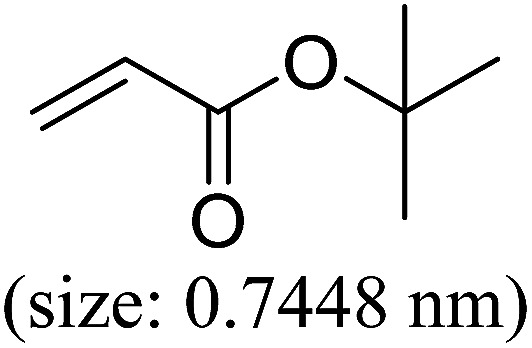	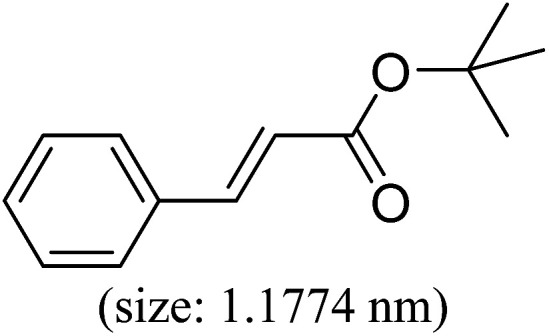	96	61
5	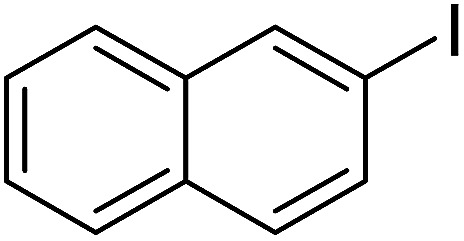	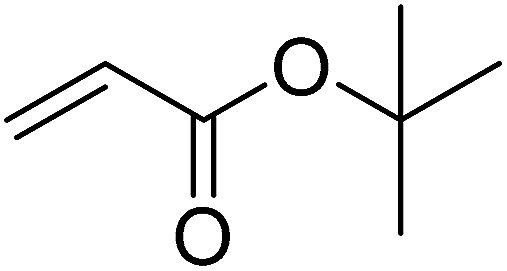	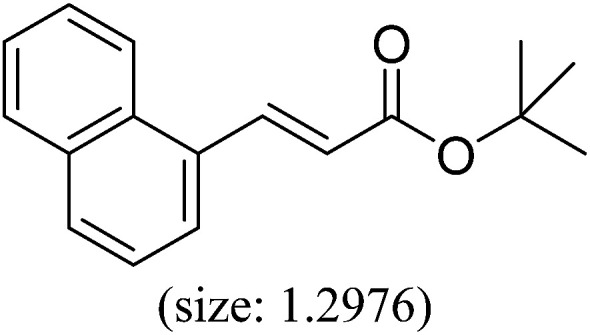	85	41
6	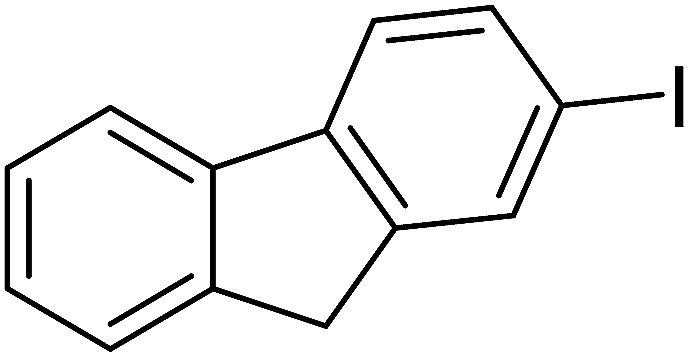	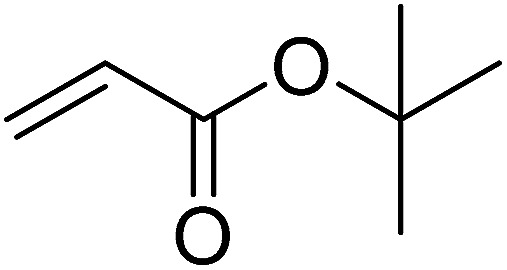	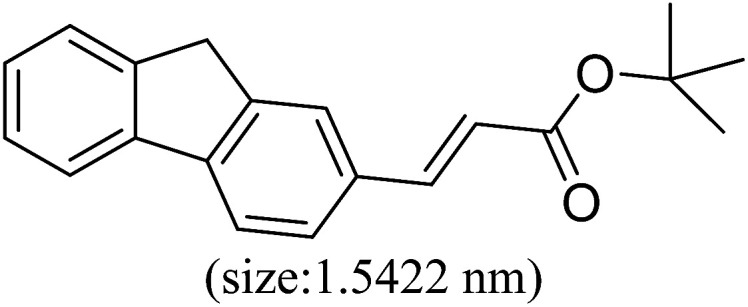	82	32

a Reaction conditions: 1 mmol aryl iodide, 2 mmol acrylate, 0.005 mmol Pd@CS membrane catalyst, 3 mmol CH_3_COOK, in 10 ml DMSO + 0.2 ml ethylene glycol solution, 110 °C, 5 h.

b GC/MS yield.

c Molecule size estimated with its bond parameters.

**Fig. 5:**
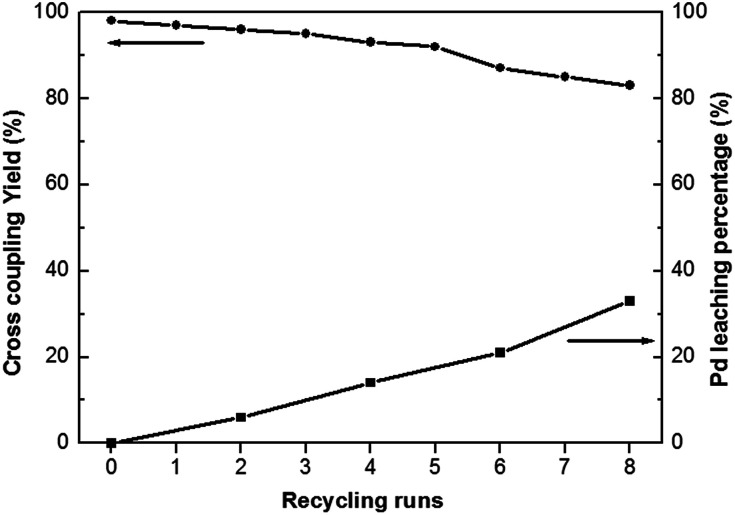
Catalytic efficiency and stability of the reused Pd^0^@CS-3 membrane catalyst.

Heterogeneous catalysts often contain the advantage of easy separation and recycling from the reaction mixture. Heck coupling reaction of iodobenzene with *n*-butyl acrylate has been employed as a model reaction to assess the stability and reusability of the Pd^0^@CS-3 membrane catalyst. As shown in Fig. 5, Pd^0^@CS-3 membrane catalyst can be recycled for more than 8 times with no significant decrease in coupling yield. The Pd leaching percentage of the recycled Pd^0^@CS-3 membrane catalyst had been monitored with ICP. It is found that about 60% of Pd is still retained in the recycled Pd^0^@CS-3 membrane catalyst after reuse of 8 times, suggesting fairly good stability.

**Fig. 6:**
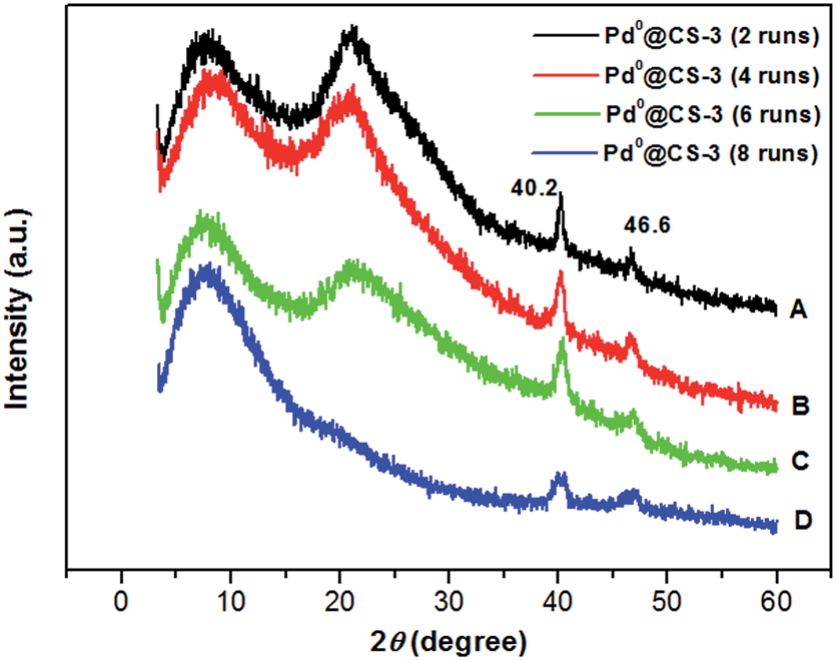
XRD patterns of the reused Pd^0^@CS-3 membrane catalyst with different recycling runs.

Examination of Fig. 6 shows the changes in the crystalline structure of the reused Pd^0^@CS-3 membrane catalyst with the increasing of the recycling runs. Characteristic diffraction peaks at 2*θ* degree of 40.2° and 46.6° of the crystal of Pd^0^ species have been detected for the reused Pd^0^@CS-3 membrane catalyst, indicating that more Pd^2+^ species have been reduced to Pd^0^ nano-particles to form perfect crystals during the catalytic reactions. The characteristic diffraction peaks of the crystal Pd^0^ species become obviously weaker after the Pd^0^@CS-3 membrane catalyst reused for 8 runs. This is mainly due to the Pd leaching during the recycling of the Pd^0^@CS-3 membrane catalyst.

**Fig. 7:**
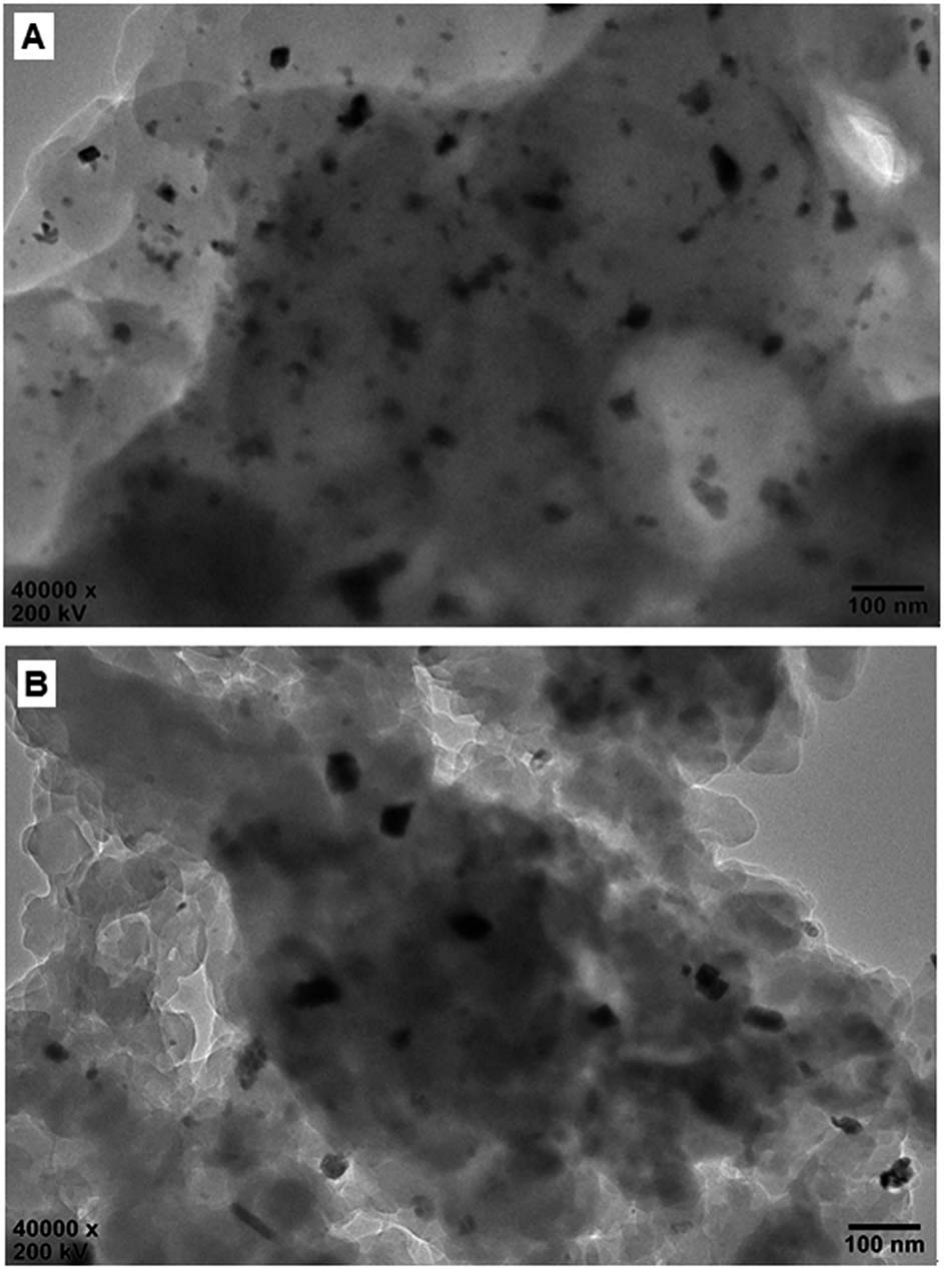
HR-TEM observation of the reused Pd^0^@CS-3 membrane catalyst with different recycling runs: (A) 4 runs; (B) 8 runs.

Fig. 7 shows the HR-TEM images of the reused Pd^0^@CS membrane catalyst. In the case of Pd^0^@CS membrane catalyst reused for 4 runs, besides plenty of Pd^0^ nano-particles in the similar size (about 5 nm) with those of the fresh Pd^0^@CS membrane catalyst (Fig. 3B), there are many Pd^0^ nanoparticle aggregates with bigger size of about 10–30 nm dispersed in the CS matrix. In the case of Pd^0^@CS membrane catalyst reused for 8 runs, the numbers of Pd^0^ nanoparticles decreases obviously, and most of the dispersed Pd^0^ nanoparticles are in bigger size of about 10–30 nm, suggesting the leaching of the small sized Pd^0^ nano-particles. These results are well in accordance with the results of XRD characterization.

**Fig. 8:**
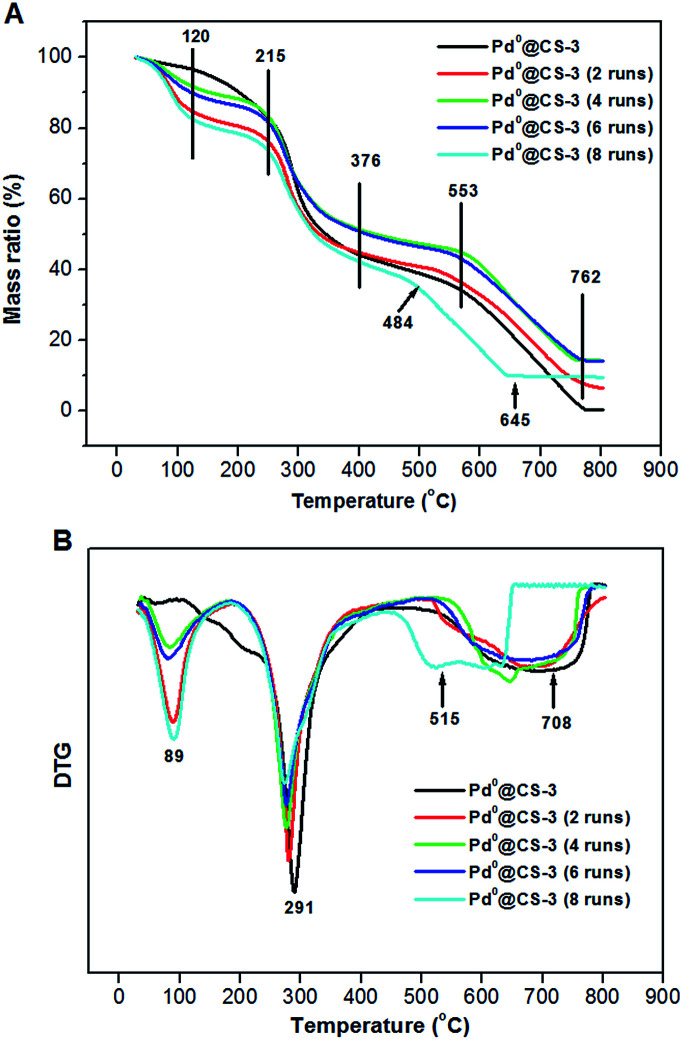
TGA curves (A) and DTG curves (B) of the reused Pd^0^@CS-3 membrane catalysts with different recycling runs.

The TGA–DTG curves of the recycled Pd^0^@CS-3 are shown in Fig. 8. For fresh Pd^0^@CS membrane catalyst, it has three weight loss stages at 50–120 °C (absorbed water evaporation), 215–376 °C (destruction of the intermolecular interactions such as hydrogen bonding and crosslinking, decomposition of chitosan macromolecules, and carbonization), and 553–762 °C (deep decomposition, carbonization, and thermal oxidation). The weight loss peak temperatures of each stage from the DTG curve of fresh Pd^0^@CS membrane catalyst was estimated as to be 89, 291, and 708 °C, respectively. Except a bit lower shifts in the corresponding weight loss peak temperatures, the Pd^0^@CS membrane catalysts reused up to 6 runs have similar weight loss stages with fresh Pd^0^@CS membrane catalyst. In the case of Pd^0^@CS membrane catalyst reused for 8 runs, the third weight loss stage and its corresponding peak temperature shifts to 484–645 °C, and 515 °C, respectively, suggesting obviously drop in the thermal stability. The decrease in thermal stability is due to the physical aging caused by prolonged use under harsh reaction conditions, such as high reaction temperature (110 °C), continuous long-time processing (reused for 8 runs), and swelling of the solvents (polar solvents corrosions), and so on.

**Fig. 9:**
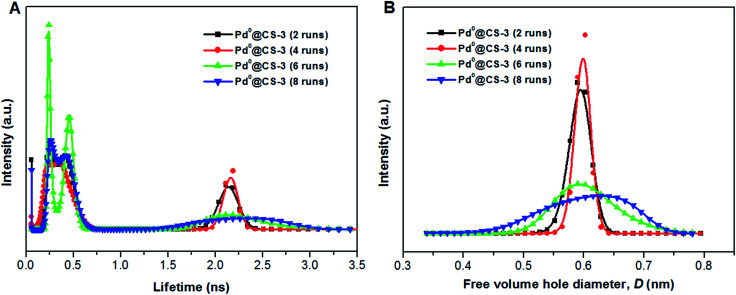
The distribution of positron annihilation lifetime (A) and free volume hole size of the reused Pd^0^@CS-3 membrane catalyst (B) analyzed by MELT program.

The mean value of lifetimes and intensities of the reused Pd^0^@CS-3 membrane catalyst are summarized in Table 4. It is found that the free volume hole size (*D*) of the reused Pd^0^@CS membrane catalyst increased as the increase of the reusing runs, suggesting a decrease in the compactness of the segments packing of CS macromolecules. On the one hand, during recycling, the Pd leaching and the formation of more Pd^0^-nanoparticles with big size will lead to a decrease of crosslinking effects of Pd^2+^. On the other hand, the CS matrix will undergo degradation for the long-time continuous processing of the recycled Pd^0^@CS-3 membrane catalyst at high temperature. The changes of the distribution of the lifetimes and free volume hole size of the reused Pd^0^@CS-3 membrane catalyst is shown in Fig. 9. It is found that the distribution peak of free volume size of the reused Pd^0^@CS-3 membrane catalyst shifts to bigger size range as the reusing runs increased. Meanwhile, the distribution peak of free volume size becomes much broader after the Pd^0^@CS-3 membrane catalyst was reused for more than 4 runs. The enlarged free volume holes must also act as the Pd leaching channels of the reused Pd^0^@CS-3 membrane catalyst. We then take similar approximate evaluation (by eqn (4)) of the mean free volume size (*D*_s_, as shown in Table 4) of the swollen reused Pd^0^@CS-3 membrane catalyst in the reaction solution. Clearly, most of the Pd^0^ nanoparticles have bigger size (as shown in HR-TEM images) than single free volume hole of the Pd^0^@CS-3 membrane catalyst. It indicates the Pd leaching is mainly achieved through a number of instantaneously connected free volume holes rather than a single free volume hole.

**Table 4:** Variation of positron annihilation lifetimes, intensities, and mean diameter of free volume holes of the reused Pd@CS-3 membranes analyzed by LT-9 program

Sample	*τ*_1_ (ns)	*I*_1_ (%)	*τ*_2_ (ns)	*I*_2_ (%)	*τ*_3_ (ns)	*I*_3_ (%)	*D* (nm)	*D*_s_ (nm)
Pd^0^@CS-3 (2 runs)	0.2430 ± 0.0044	53.8 ± 1.3	0.4780 ± 0.010	38.0 ± 1.2	2.288 ± 0.013	8.2 ± 0.3	0.6198 ± 0.0020	1.0041 ± 0.0032
Pd^0^@CS-3 (4 runs)	0.2217 ± 0.0028	60.4 ± 1.0	0.4844 ± 0.009	32.4 ± 1.0	2.375 ± 0.014	7.2 ± 0.2	0.6340 ± 0.0021	1.0271 ± 0.0034
Pd^0^@CS-3 (6 runs)	0.2415 ± 0.0039	63.9 ± 1.0	0.5272 ± 0.007	29.3 ± 0.8	2.425 ± 0.011	6.8 ± 0.2	0.6418 ± 0.0018	1.0397 ± 0.0029
Pd^0^@CS-3 (8 runs)	0.2409 ± 0.0046	65.4 ± 1.1	0.5171 ± 0.007	28.7 ± 0.9	2.450 ± 0.013	5.9 ± 0.3	0.6458 ± 0.0021	1.0461 ± 0.0034

## 4. Conclusions

In this study, we have combined the positron annihilation lifetime spectrum and other structural characterization methods to elucidate the correlations between the microstructure and the catalytic performances of Pd@CS membrane heterogeneous catalysts. It was found that the *in situ* reduction of Pd^2+^ into Pd^0^ nanoparticles in the interstices of CS macromolecules was effectively entrapped in the matrices of Pd^0^@CS membrane, which still allowed the diffusion of reactant and product molecules through the matrices. We demonstrated that molecules transport channels of Pd^0^@CS membrane catalyst in the reaction at high temperature was through a number of instantaneously connected free volume holes rather than a single free volume hole. The findings in this study may be useful to further understand the catalytic mechanism in molecular level of transition metals supported on biopolymers heterogeneous catalysts, in pursuit of rational development of efficient catalysis reaction system.

## Conflicts of interest

There are no conflicts to declare.

## Supplementary Material
